# Problems and Progress regarding Sex Bias and Omission in Neuroscience Research

**DOI:** 10.1523/ENEURO.0278-17.2017

**Published:** 2017-11-09

**Authors:** Tyler R. Will, Stephanie B. Proaño, Anly M. Thomas, Lindsey M. Kunz, Kelly C. Thompson, Laura A. Ginnari, Clay H. Jones, Sarah-Catherine Lucas, Elizabeth M. Reavis, David M. Dorris, John Meitzen

**Affiliations:** 1 Department of Biological Sciences, North Carolina State University, Raleigh, NC; 2 Graduate Program in Zoology, North Carolina State University, Raleigh, NC; 3 W.M. Keck Center for Behavioral Biology, North Carolina State University, Raleigh, NC; 4 Graduate Program in Physiology, North Carolina State University, Raleigh, NC; 5 Department of Psychology, North Carolina State University, Raleigh, NC; 6 Center for Human Health and the Environment, North Carolina State University, Raleigh, NC; 7 Comparative Medicine Institute, North Carolina State University, Raleigh, NC

**Keywords:** Animal models, journals, neuroscience, sex bias, sex omission

## Abstract

Neuroscience research has historically ignored female animals. This neglect comes in two general forms. The first is sex bias, defined as favoring one sex over another; in this case, male over female. The second is sex omission, which is the lack of reporting sex. The recognition of this phenomenon has generated fierce debate across the sciences. Here we test whether sex bias and omission are still present in the neuroscience literature, whether studies employing both males and females neglect sex as an experimental variable, and whether sex bias and omission differs between animal models and journals. To accomplish this, we analyzed the largest-ever number of neuroscience articles for sex bias and omission: 6636 articles using mice or rats in 6 journals published from 2010 to 2014. Sex omission is declining, as increasing numbers of articles report sex. Sex bias remains present, as increasing numbers of articles report the sole use of males. Articles using both males and females are also increasing, but few report assessing sex as an experimental variable. Sex bias and omission varies substantially by animal model and journal. These findings are essential for understanding the complex status of sex bias and omission in neuroscience research and may inform effective decisions regarding policy action.

## Visual Abstract

**Figure:**
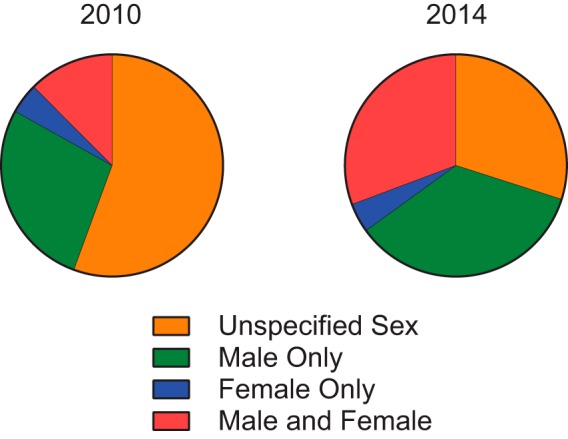

## Significance Statement

Neuroscience research has historically favored the use of male over female animals or often ignored animal sex. Recognition of this sex bias and omission has spurred fierce debate and study, including new regulatory policies and scientific findings. Here we further probe this phenomenon by conducting the largest-ever analysis of neuroscience research articles for sex bias and omission. We show that sex bias is still present, and that sex omission is declining. The extent of sex bias and omission varies widely by animal model and journal. These results produce key implications for research conduct, regulatory policies, and scientific culture by revealing the still-present but complex nature of sex bias and omission.

## Introduction

Neuroscience research has historically demonstrated sex bias, in this case favoring the use of male over female research animals, and sex omission, which is the lack of reporting research animal sex (Berkley, 1992; Mogil and Chanda, 2005; Beery and Zucker, 2011; Shansky and Woolley, 2016). Although neuroscience is not the only biomedical discipline exhibiting sex bias, Beery and Zucker (2011) demonstrated that neuroscience, pharmacology, physiology, and endocrinology exhibited the largest sex biases in research animal use out of 10 analyzed disciplines. Collectively, this phenomenon of discipline-specific sex bias has generated fierce debate, resulting in awareness campaigns, studies, regulatory policies, and position commentaries (Becker et al., 2005, 2016; Clayton and Collins, 2014; Fields, 2014; Johnson et al., 2014; McCullough et al., 2014; Ruigrok et al., 2014; Yoon et al., 2014; Cahill and Aswad, 2015; Klein et al., 2015; McCarthy, 2015; Park et al., 2015; Richardson et al., 2015; Eliot and Richardson, 2016; Guizzetti et al., 2016; Maney, 2016; Mogil, 2016; Panzica and Melcangi, 2016; Tannenbaum et al., 2016; Zakiniaeiz et al., 2016; Brooks and Clayton, 2017; Duchesne et al., 2017; Joel and McCarthy, 2017; Karp et al., 2017; McEwen and Milner, 2017; Miller et al., 2017). Many authors argue that it is vital to document experimental animal sex, and to thoughtfully select and justify the sex of experimental animals. Important for this discussion, and especially for the implementation and evaluation of regulatory policies, is the evaluation of sex bias and omission in the neuroscience research literature. Here we provide these data by testing the hypotheses that sex bias and omission still persist in the neuroscience literature, that studies employing both males and females neglect sex as an experimental variable, and that sex bias and omission vary by rodent species and journal origin. To accomplish this, our team of 11 trained curators assessed all research articles using rats or mice published from 2010 to 2014 in the following journals: *Journal of Neuroscience* (*J. Neurosci.*), *Journal of Neurophysiology* (*J. Neurophysiol.*), *Nature Neuroscience* (*Nat. Neurosci.*), *Neuron*, *Nature*, and *Science*. These journals were chosen given their prominence in the neuroscience field and also to align with previous studies (Beery and Zucker, 2011; Shansky and Woolley, 2016). A comprehensive approach to article selection was undertaken to decrease sampling bias within the analyzed journals, and research articles were analyzed given that this is the final common output of academic neuroscience research.

## Materials and Methods

### Inclusion criteria and coding of articles

Articles were analyzed from 2010 to 2014 from the following journals: *J. Neurosci.*, *J. Neurophysiol.*, *Nat. Neurosci.*, *Neuron*, *Nature*, and *Science*. A team of 11 trained curators [8 females, 3 males; Assessing Rodent Sex in Neuroscience Literature (ARSiNL) team] examined all articles published per year within the targeted journals. Trained curators were used because the divergent and extensive vocabulary used to describe animal sex and its treatment as an experimental variable make automated text-mining approaches challenging. Articles were first determined to be primary research articles by the curators. Following previously published studies (Berkley, 1992; Sechzer et al., 1994; Mogil and Chanda, 2005; Beery and Zucker, 2011; Yoon et al., 2014; Shansky and Woolley, 2016), reviews, editorials, and similar nonprimary research articles were excluded from analysis. Articles were then analyzed for neuroscience relevance. Articles from *J. Neurosci.*, *J. Neurophysiol.*, *Nat. Neurosci.*, and *Neuron* were automatically accepted as neuroscience relevant. A broad inclusion criterion was employed for articles from *Nature* and *Science*: articles in these journals were included for analysis if the article topic encompassed any aspect of the central or peripheral nervous system, ranging from the molecular to behavioral level of analysis. In all journals, articles using fetal animals and primary neuron cultures were included in the overall analysis as in a previous study (Taylor et al., 2011), given that cells express chromosomal sex (XX or XY) and that sex differences have been detected even at the embryonic stage and in primary neuron culture. These inclusion criteria identified 13,857 primary research neuroscience articles. Articles were then coded for species. Species categories were mouse, rat, and other. Articles using other species were excluded from further analysis, resulting in a pool of 6636 neuroscience articles that employed rats or mice; 2611 articles employed rats, and 4221 articles employed mice. Articles using a rat or mouse and another species were included in analysis, with the nonrodent portion of the article excluded from analysis. Articles using both mice and rats were included in analysis (196 articles). Articles using both mice and rats were included in both the mice and rat categories, but only counted once in analyses that combined mice and rat datasets. The reason for focusing the study on the analysis of articles employing mice or rats is further explained in Discussion.

Articles were then analyzed for research animal sex. Sex categories were male, female, no sex reported, and male and female. Articles containing both male and females were further subdivided into those wherein biological sex was not considered as an experimental variable and those wherein biological sex was considered an experimental variable. Articles were considered to have addressed sex as a biological variable if any formal statistical comparison or assertion of such a comparison of males and females was performed, including if the data or analysis was not shown, and including whether sex differences were detected or not. Very few articles reported data disaggregated by sex but did not perform or assert to have performed a statistical comparison. These articles were coded as not having addressed sex as an experimental variable since there was no comparison. Intra- and intercurator error rates were assessed, with the rates being 0% and 7%, respectively. Experimental power was not assessed. When distinct experiments within an article employed different sexes, articles were considered male/female with biological sex not considered an experimental variable, following a previous study (Beery and Zucker, 2011).

### Statistics

Experiments were analyzed via linear regression and ANCOVA (Prism version 6.07, GraphPad Software). *P* values <0.05 were considered a priori as significant. Data are presented as percentages or absolute proportions. Further statistical information is presented in Table 1.

**Table 1.** Details of statistical analysis

Figure	Data structure	Type of test	Confidence intervals
1*B*	Normal distribution	Linear regression	–4.389 to 3.178
2*A*	Normal distribution	Linear regression	Male only: 0.8086 to 3.575; female only: –0.5272 to 1.069; male and female: 1.277 to 8.273; unspecified sex: –12.64 to –1.834
2*B*	Normal distribution	Linear regression	Male only: 1.105 to 2.797; female only: –0.5271 to 1.073; male and female: 2.294 to 8.554; unspecified sex: –12.16 to –3.138
2*C*	Normal distribution	Linear regression	Male only: 1.197 to 6.233; female only: –0.1067 to 0.8067; male and female: –1.040 to 7.242; unspecified sex: –13.97 to –0.3654
3	Normal distribution	Linear regression	–6.877 to 2.767
5*A*	Normal distribution	Linear regression, ANCOVA	*J. Neurophysiol.*: –10.05 to –1.779; *J. Neurosci.*: –16.76 to 0.5671; *Nature*: –18.14 to –8.113; *Nat. Neurosci.*: –21.17 to –1.192; *Neuron*: –4.831 to –1.845; *Science*: –8.771 to 12.49
5*B*	Normal distribution	Linear regression, ANCOVA	*J. Neurophysiol.*: 0.03390 to 5.726; *J. Neurosci.*: –0.8596 to 11.99; *Nature*: 1.781 to 22.73; *Nat. Neurosci.*: –1.290 to 9.552; *Neuron*: –0.5016 to 5.360; *Science*: –8.859 to 9.249
5*C*	Normal distribution	Linear regression, ANCOVA	*J. Neurophysiol.*: –0.4495 to 6.815; *J. Neurosci.*: 0.3678 to 3.688; *Nature*: –5.311 to 7.824; *Nat. Neurosci.*: –0.6518 to 13.34; *Neuron*: –1.601 to 3.571; *Science*: –11.46 to 8.715
5*D*	Normal distribution	Linear regression, ANCOVA	*J. Neurophysiol.*: –0.5273 to 0.2373; *J. Neurosci.*: –2.637 to 1.863; *Nature*: –2.637 to 1.862; *Nat. Neurosci.*: –1.188 to 2.426; *Neuron*: –2.402 to 2.252; *Science*: –4.550 to 3.180

Confidence intervals for linear regressions indicate the 95% confidence interval surrounding the slope. *R* ^2^ and other relevant statistics are reported in Results. ANCOVA, analysis of covariance.

## Results

**Figure 1.**
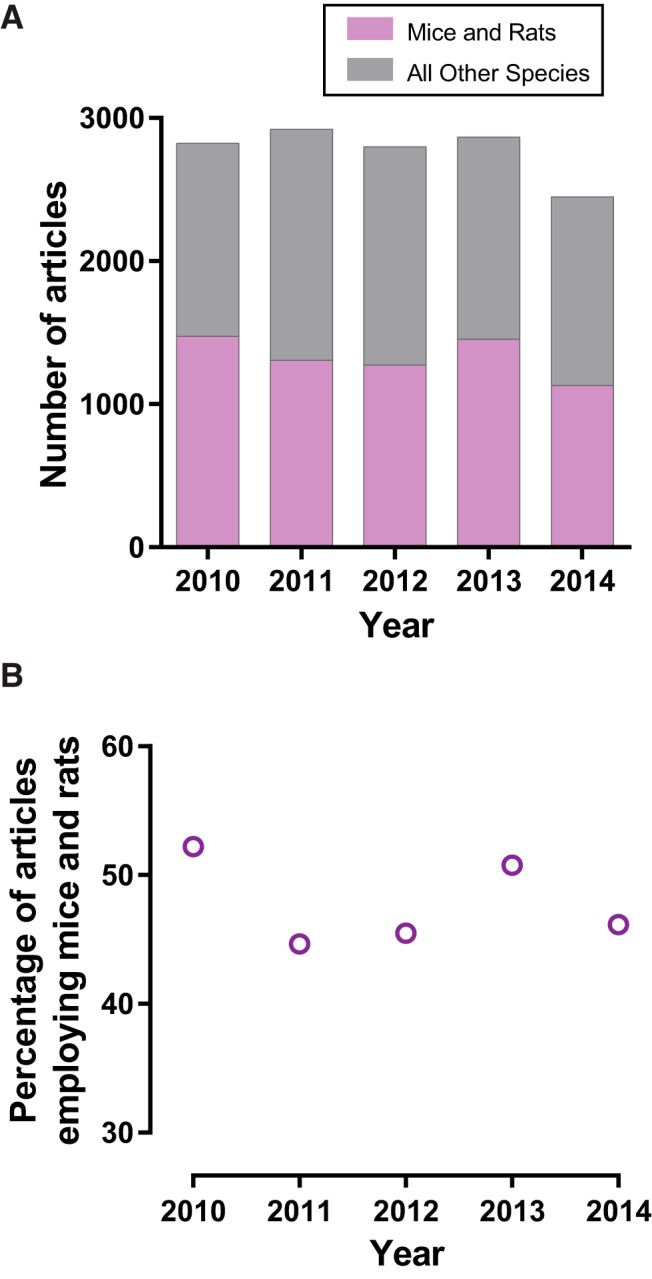
Articles using mice and rats are a significant and stable proportion of the neuroscience literature. ***A***, From 2010 to 2014, 13,857 neuroscience research articles were published by the *J. Neurosci.*, *J. Neurophysiol.*, *Nat. Neurosci.*, *Neuron*, *Science*, and *Nature* (gray bar). Of these articles, 6,636 used rats or mice and were further analyzed (purple bar). The total number of articles using mice and rats was consistently distributed across years. ***B***, The percentage of articles using rats or mice remained fairly constant across years.

Our research article inclusion criteria resulted in an initial pool of 13,857 neuroscience research articles. Of these articles, 6636 used rats or mice and were further analyzed for sex bias and omission (Fig. 1*A*). The percentage of articles using rats or mice remained fairly constant across years, with a calculated linear regression finding no correlation between the percentage of articles using mice and rats and year (Fig. 1*B*; slope −0.61, *r* ^2^ = 0.08, *p* > 0.05). From these findings we concluded that articles using mice and rats are a significant and stable proportion of the neuroscience literature.

### Sex omission is decreasing but sex bias remains present

**Figure 2.**
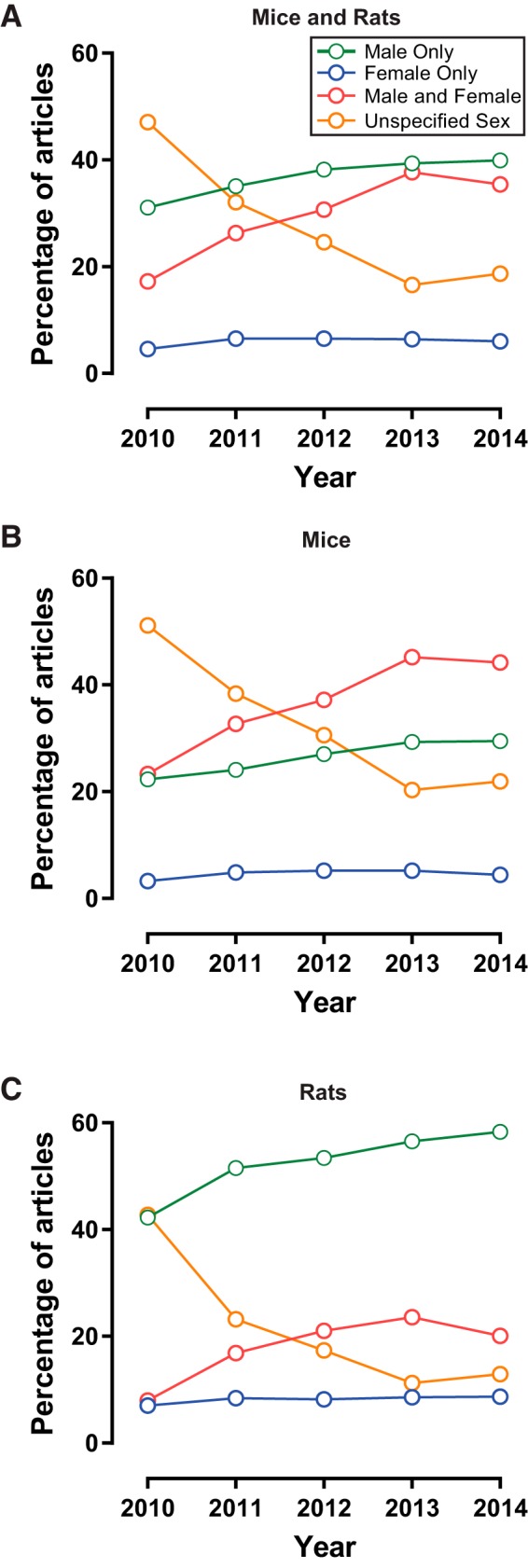
Sex omission is decreasing but sex bias remains present, with different patterns observed in articles using mice versus those using rats. Articles were categorized as either not reporting sex (orange) or reporting both males and females (red), only males (green), or only females (blue). ***A***, All articles, using both mice and rats. Articles not reporting animal sex decreased from 2010 to 2014. Articles using only male animals increased from 2010 to 2014, comprising the largest proportion of articles by 2011. Articles reporting the use of both male and female animals also increased over time, nearing but not overtaking the percentage of articles using only males by 2013. Articles using only female animals remained stable and low. ***B***, Mice. Articles not reporting mice sex decreased from 2010 to 2014. Articles reporting the use of both male and female mice increased over time and comprised the largest proportion of articles by 2012. Articles using only male mice increased from 2010 to 2014. Articles using only female mice remained stable and low. ***C***, Rats. Articles not reporting rat sex decreased from 2010 to 2014. Article using only male rats increased from 2010 to 2014 and comprised the largest proportion of articles by 2011. Articles reporting the use of both male and female rats increased from 2010 to 2014, but were a much smaller proportion of the dataset than articles using only male rats. Articles using only female rats remained stable and low.

Articles using rats and mice were then analyzed to determine how animal sex was reported (Fig. 2*A*). Articles were categorized as either not reporting sex, or reporting both males and females, only males, and only females. The percentage of articles not reporting sex decreased from 47% in 2010% to 19% in 2014 (slope −7.24, *r*^2^ = 0.86, *p* < 0.03). The percentage of articles reporting both male and female animals increased from 17% in 2010% to 38% in 2013 and plateaued at 35% in 2014 (slope 4.78, *r*^2^ = 0.86, *p* < 0.03). Articles reporting only males increased from 31% in 2010% to 40% in 2014 (slope 2.19, *r*^2^ = 0.89, *p* < 0.02). The percentage of articles reporting only female animals remained stable and low throughout the assessed period, ranging from 5% in 2010% to 6% in 2014 (slope 0.27, *r*^2^ = 0.28, *p* > 0.05). Overall, these results indicate that sex omission is decreasing and that sex bias remains present over the assessed period, with articles reporting the sole use of males not only comprising the largest proportion of published articles, but also continuing to increase across years.

### Sex bias and omission vary considerably by animal model

We next tested the hypothesis that sex bias and omission vary by animal model. Many more articles used mice (4221) than rats (2611), which could potentially influence a dataset incorporating both species. In both mice (Fig. 2*B*) and rats (Fig. 2*C*), the percentage of articles not reporting sex decreased between 2010 and 2014 (mice: slope −7.65, *r*^2^ = 0.91, *p* < 0.02; rats: slope −7.17, *r*^2^ = 0.79, *p* < 0.05). In mice, articles reporting both males and females increased over time, and comprised the largest proportion of published articles by 2012, and reached 44% by 2014 (slope 5.42, *r*^2^ = 0.91, *p* < 0.02). Articles reporting only males also increased, but to a lesser extent, from 22% in 2010% to 29% in 2014 (slope 1.95, *r*^2^ = 0.95, *p* < 0.006). The percentage of articles reporting only females remained low and stable, ranging from 3% in 2010% to 4% in 2014 (slope 0.27, *r*^2^ = 0.28, *p* > 0.05). In contrast to mice, for articles using rats the percentage reporting males dominated the distribution, ranging from 42% in 2010% to 58% in 2014 (slope 3.72, *r*^2^ = 0.88, *p* < 0.02), and showed a substantially different *y*-intercept compared with mice (rats: –7422; mice: –3899) There was also an absolute increase in the percentage of articles reporting both males and females from 8% in 2010% to 20% in 2014, but this did not reach significance because of the relatively stable percentages from 2012 to 2014 (21%, 24%, and 20%, respectively; slope 3.10, *r*^2^ = 0.65, *p* > 0.05). Similar to mice, the percentage of articles reporting only female rats remained low, ranging from 7% in 2010 to 9% in 2014 (rats: slope 0.35, *r*^2^ = 0.66, *p* > 0.05). These findings demonstrate that articles using different species show important distinctions that diverge across time. Although both species show decreases in sex omission, by 2014 sex is less likely to be reported in mice studies compared to rat studies. Regarding sex bias, by 2014 the majority of rat studies report the use of only males. A substantial and increasing percentage of mice studies also report the use of only males; however, a larger proportion of mice studies report the use of both males and females.

### Most research articles incorporating both males and females do not assess sex as an experimental variable

**Figure 3.**
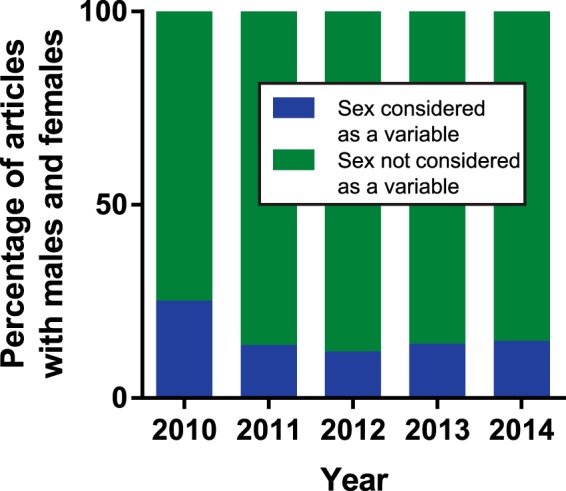
The vast majority of articles using both male and female animals do not report analyzing sex as an experimental variable. Articles using both male and female animals were evaluated for any formal statistical test or statement that data from males and females were compared, regardless of outcome and whether or not data were reported. The overall percentage of articles incorporating sex as an experimental variable remained low and relatively stable from 2011 to 2014 (∼14%), after a noticeable decrease from the year 2010 (25%).

Although it is promising that more articles are reporting the use of both males and females, these articles do not necessarily consider sex as an experimental variable. This phenomenon was first documented by Beery and Zucker (2011), who found that only ∼20% of neuroscience studies that used both sexes actually analyzed data by sex. We thus tested whether articles using both males and females reported any statistical test or statement indicating that data from males and females were compared, whether a sex difference or similarity was detected. Our analysis found that the vast majority of articles did not report considering sex as an experimental variable, although both males and females were included in the study (Fig. 3). Depending on the year, only 12%–25% of assessed studies included any indicator that data from males and females were compared. The overall percentage of articles incorporating sex as an experimental variable remained relatively stable from 2011 to 2014, after a substantial decrease between the years 2010 (25%) and 2011 (14%; Fig. 3; slope –2.055, *r* ^2^ = 0.38, *p* > 0.05). These data show that although there is increased documentation of the use of males and females, most studies still do not report analyzing sex as an experimental variable.

### Sex bias and omission varies between journals

**Figure 4.**
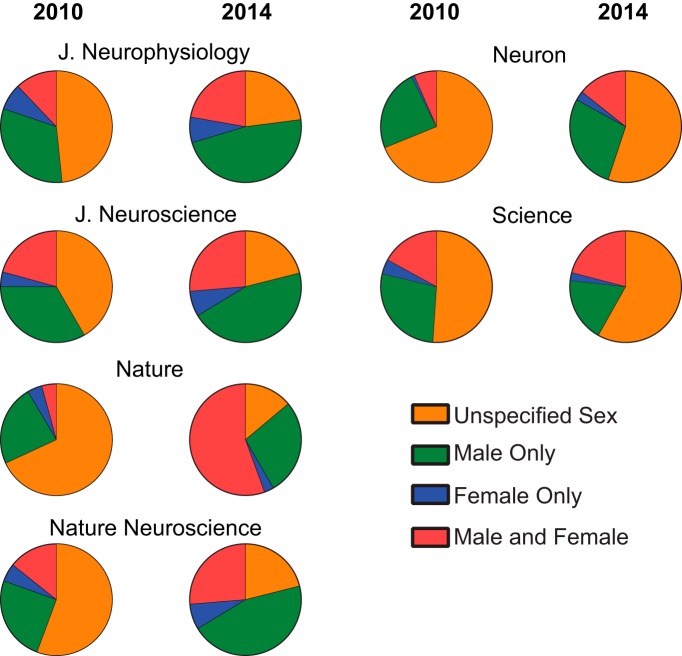
Sex omission and bias differ by journal and change from 2010 to 2014. Articles were analyzed from the following journals: *J. Neurosci.*, *J. Neurophysiol.*, *Nat. Neurosci.*, *Neuron*, *Science*, and *Nature*. Four of the six journals showed large decreases in sex omission. Of this group, *Neuron* showed the smallest decrease, beginning with 69% of articles not reporting sex in 2010, decreasing to 55% in 2014. In contrast, one journal, *Science*, showed an increase in the percentage of articles not reporting sex, rising from 51% in 2010% to 58% in 2014.

**Figure 5.**
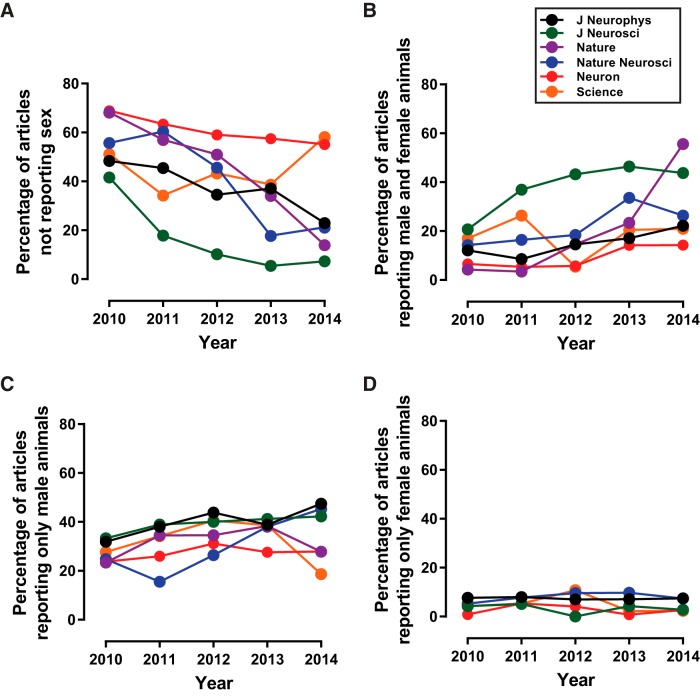
Patterns of sex omission and bias markedly differ across years by journal. ***A***, Articles not reporting sex. The percentage of articles not reporting sex decreased in five of six journals. The percentage of articles not reporting sex increased in the journal *Science*. The journals *Science* and *Neuron* showed high percentages of articles not reporting sex. ***B***, Articles reporting both males and females. Most journals show increased percentages of articles reporting both males and females, although different patterns occur across time. ***C***, Articles reporting only males. ***D***, Articles reporting only females. The percentage of articles reporting the sole use of female animals remained stable and low in all journals. Green, *J. Neurosci.*; black, *J. Neurophysiol.*; blue, *Nat. Neurosci.*; red, *Neuron*; orange, *Science*; purple, *Nature*.

An important facet of the analysis presented thus far is that it pooled articles across six different journals. This provides the advantage of a broad sampling of the neuroscience literature. One limitation is that scientific journals may have differing policies and customs regarding methods of documentation, including the requirement of reporting sex. This may create differences between journals in the percentage of articles reporting varying categories of animal sex. To address this question, articles were analyzed by their journal of origin, including *J. Neurophysiol.* (848 articles), *J. Neurosci.* (4105 articles), *Nature* (243 articles), *Nat. Neurosci.* (582 articles), *Neuron* (649 articles), and *Science* (209 articles; Fig. 4). Journals differed in the percentages of articles not reporting sex from 2010 to 2014 (Fig. 5*A*; F_(5,18)_ = 5.42, *p* < 0.004). In five of the six journals, the percentage of articles not reporting sex decreased between 2010 and 2014, although there were varying degrees of change in magnitude between journals (Fig. 5*A*; *J. Neurophysiol.*: slope –5.92, *r*^2^ = 0.87, *p* < 0.02; *J. Neurosci.*: slope –8.10, *r*^2^ = 0.75, *p* = 0.059; *Nature*: slope –13.13, *r*^2^ = 0.96, *p* < 0.004; *Nat. Neurosci.*: slope –11.18, *r*^2^ = 0.81, *p* < 0.04; *Neuron*: slope –3.34, *r*^2^ = 0.94, *p* < 0.006). Of this group, *Neuron* showed the least overall change in magnitude, beginning with 69% of articles not reporting sex in 2010, decreasing to only 55% in 2014. In contrast, one journal, *Science*, showed a surprising increase in the percentage of articles with undocumented sex in 2014 compared to earlier years, increasing from 51% in 2010% to 58% in 2014 (Fig. 5*A*; slope 1.86, *r*^2^ = 0.09, *p* > 0.05).

Journals also differed in the percentages of articles reporting male and female animals (Fig. 5*B*; F_(5,18)_=3.78, *p* < 0.02), with most journals showing varying patterns of increased percentages from 2010 to 2014 (*J. Neurophysiol.*: slope 2.88, *r*^2^ = 0.78, *p* < 0.05; *J. Neurosci.*: slope 5.57, *r*^2^ = 0.72, *p* = 0.07; *Nature*: slope 12.26, *r*^2^ = 0.82, *p* < 0.04; *Nat. Neurosci.*: slope 4.13, *r*^2^ = 0.66, *p* = 0.09; *Neuron*: slope 2.43, *r*^2^ = 0.70, *p* = 0.07; *Science*: slope 0.20, *r*^2^ = 0.00, *p* > 0.05). Journals did not differ in the overall change/slope of the percentage of articles reporting only males (Fig. 5*C*; F_(5,18)_ = 1.86, *p* > 0.05). However, elevations between journals significantly differed (F_(5,23)_ = 3.09, *p* < 0.03), and select journals showed changes across time in the percentage of articles reporting only males (*J. Neurophysiol.*: slope 3.18, *r*^2^ = 0.72, *p* = 0.07; *J. Neurosci.*: slope 2.03, *r*^2^ = 0.83, *p* < 0.04; *Nature*: slope 1.26, *r*^2^ = 0.11, *p* > 0.05; *Nat. Neurosci.*: slope 6.34, *r*^2^ = 0.74, *p* = 0.06; *Neuron*: slope 0.99, *r*^2^ = 0.33, *p* > 0.05; *Science*: slope –1.37, *r*^2^ = 0.06, *p* > 0.05). Similarly, journals also did not differ in the overall change in the percentage of articles reporting only females (Fig. 5*D*; F_(5,18)_ = 0.36, *p* > 0.05), but likewise showed a significant difference in elevation (F_(5,23)_ = 5.30, *p* < 0.003). No individual journals showed changes across time in the percentage of articles reporting only females (*J. Neurophysiol.*: slope –0.15, *r*^2^ = 0.33, *p* > 0.05; *J. Neurosci.*: slope –0.39, *r*^2^ = 0.09, *p* > 0.05; *Nature*: slope –0.39, *r*^2^ = 0.09, *p* > 0.05; *Nat. Neurosci.*: slope 0.62, *r*^2^ = 0.28, *p* > 0.05; *Neuron*: slope –0.08, *r*^2^ = 0.00, *p* > 0.05; *Science*: slope –0.69, *r*^2^ = 0.10, *p* > 0.05).

## Discussion

The key finding of this study is that substantial progress has been made in the reduction of sex omission, but that male sex bias remains a persistent and perhaps even intensifying phenomenon in the neuroscience literature. Complementing this general finding, we find that sex omission and bias vary considerably between journal and animal model. This indicates that though it is accurate to state that sex omission and bias is a generalizable phenomenon across neuroscience research, the extent and nature of omission and bias should be carefully documented and defined to achieve maximum practical utility. For example, levels of sex bias and omission differ markedly between studies employing rats than those employing mice. This finding explains a discrepancy between a prior study that detected weaker sex bias and omission but limited its automated text mining analysis to biomedical studies that employed mice (Flórez-Vargas et al., 2016), compared with studies that employed trained curators but analyzed biomedical and neuroscience studies that employed multiple model animals (Berkley, 1992; Sechzer et al., 1994; Mogil and Chanda, 2005; Beery and Zucker, 2011; Yoon et al., 2014; Shansky and Woolley, 2016).

This study detected a distinct shift in sex omission and bias across time. During the years 2010–2011, we detected similar levels of sex omission and male sex bias in neuroscience articles as reported by previous studies analyzing smaller data sets, providing important validation (Beery and Zucker, 2011; Shansky and Woolley, 2016). Sex omission and sex bias then markedly change during 2011–2014. During this time period, sex omission dramatically decreased, indicating significant progress in documenting research animal sex. However, as of 2014, more than 20% of all research articles still failed to report animal sex, which we consider an unacceptably high number for an essential experimental component. From a broader perspective, if such a basic detail as animal sex is omitted, other methods that may or may not seem obscure but are necessary for successful replication may also not be included in the methods section of manuscripts (Thigpen et al., 2013; Freedman et al., 2017).

Regarding male sex bias, reports of the sole use of males increased, most predominantly in rats, but also in mice. Furthermore, even when studies used both males and females, few reported incorporating sex as an experimental variable. Collectively, our data indicate that sex bias remained present and perhaps even intensified during 2010–2014, despite awareness campaigns and other efforts. Remarkably, these measured decreases in sex omission and increases in male sex bias occurred before the implementation of the National Institute of Health (NIH) Sex as a Biological Variable (SABV; NOT-OD-15-102) regulatory policy, which went into effect on January 25, 2016 (Clayton and Collins, 2014). Thus, the dataset produced by our study may prove useful for empirically evaluating the general success of SABV and similar efforts, though our study was not explicitly designed to assess article compliance with specific aspects of the SABV or any other funding agency mandate. Future studies intending to assess the impact of SABV should evaluate the success of specific aspects of SABV requirements. For instance, one subtle but relevant aspect of SABV is the requirement to prospectively develop a research design that, at a minimum, reports data disaggregated by sex without requiring a statistical test evaluating sex as an experimental variable (NIH Guide Notice NOT-OD-15-102). The design of the current study does not differentiate between studies that report data disaggregated by sex with no comparison versus studies that report aggregate sex data with no comparison. Anecdotally, our curators found very few articles that reported data disaggregated by sex but that did not perform or assert to have performed a statistical comparison by sex.

Other aspects of SABV may also be relevant to the design of future studies assessing the effect of SABV. These aspects may include the presence of justification for single-sex studies or, if both sexes are used, whether the experimental design/analysis is sufficiently powered to detect robust sex differences. Importantly, SABV is not the only relevant funding agency policy that may impact sex omission and bias in the neuroscience literature. For example, the Canadian Institutes of Health Research is a signature on the Government of Canada’s Health Portfolio Sex- and Gender-Based Analysis Policy and has detailed criteria for how to evaluate sex and gender that differs from that outlined by SABV. Because the exact policy requirements regarding biological sex vary by funding agency, future studies will need to be a priori designed to either directly assess specific funding agency policies (and whether these policies even apply to a particular research study), or generally assess sex omission and bias in the neuroscience literature regardless of research article funding source.

One aspect of the current study is that analysis was restricted to research articles using mice or rats. Articles using mice and rats were analyzed in the current work for the following four reasons. First, the wide availability of rats and mice concomitant with an abundance of research protocols and external secondary sex characteristics more easily enables the analysis of both male and female animals. Second, rats and mice have many documented sex differences in brain and behavior. Third, examination of mice and rats aligns the findings of the current study with previous work that analyzed mice or rats (Mogil and Chanda, 2005; Flórez-Vargas et al., 2016; Shansky and Woolley, 2016) and nonhuman mammals (Beery and Zucker, 2011). Fourth, a previous study indicated that mice and rats were by far the predominant species reported in neuroscience research articles (Beery and Zucker, 2011). Beery and Zucker (2011) reported that more than 85% of neuroscience research articles employed mice or rats, a much higher percentage than that detected by the current study (∼48%; Fig. 1*A*). Three possibilities may contribute to this large difference between studies in the measured proportion of research articles using mice and rats. The first possibility is differences in journal selection. Compared to the current study, Beery and Zucker (2011) analyzed an overlapping but different suite of journals representing the neuroscience discipline: *J. Neurosci.*, *Neuroscience*, *The Journal of Comparative Neurology*, and *Nat. Neurosci.*. Given that two of these journals were included in the analysis of the current study, we believe that journal selection is not likely a major influence. The second possibility regards article sampling, in that the current study analyzed a much larger number of research articles than Beery and Zucker (2011). The third possibility may be how the percentage of rat and mouse studies was calculated. Beery and Zucker (2011) used only nonhuman studies to calculate the percentage of mice and rat studies in the neuroscience literature, whereas the current study included both nonhuman and human studies. We favor this last possibility as the most likely explanation. We note that the exclusion of animals other than rats and mice from the current study was not because we consider these species (including humans) unimportant for neuroscience research. Given the finding of this study that the majority of neuroscience research articles involves work in species other than mice and rats (Fig. 1*A*), scientists from both contemporary and earlier generations likely also share this assessment (Beach, 1950; Krebs, 1975; Brenowitz and Zakon, 2015; Remage-Healey et al., 2017). Indeed, our study is the first to detect that sex bias and omission varies across any species of research animal. Based on this critical finding, future studies should address the intersection of species and sex by directly testing whether sex bias and omission vary across research animals beyond mice and rats.

Another novel and central finding of this study was the considerable variability in sex omission across journals. Because our study was not designed to elucidate the etiology of differences in sex omission between journals, it will be an important next step to understand why some journals exhibit relatively low sex omission and others do not. One possibility is differences in culture and practice between neuroscience subfields. A second possibility regards journal adoption and enforcement of relevant editorial policies, which were in flux during the assessed time period. Consistent with this possibility, beginning in 2012, *J. Neurophysiol.*, and more broadly all journals published by the American Physiologic Society (Miller, 2012), asked authors to include the sex of research animals, cells, and other biological materials. Journals published by the American Physiologic Society also recommend that authors apply the relevant portions of the “Animals in Research: Reporting *In Vivo* Experiments” (ARRIVE) guidelines (Kilkenny et al., 2010). ARRIVE guidelines cover many aspects of experimental methodology, including biological sex, in an attempt to enhance reproducibility.

The time period of 2013–2014 may prove to be a pivotal point for the reporting of not only animal sex, but other methodological details as well. Building on earlier workshops such as the “Sex-Specific Reporting of Scientific Research” hosted by the Office of Research on Women’s Health of the National Institutes of Health (Wizemann, 2012), in June 2014, a conference including representatives of the U.S. National Institutes of Health, the American Association for the Advancement of Science, and editors representing >30 scientific journals, established the Principles and Guidelines in Reporting Preclinical Research (McNutt, 2014; Nature, 2014; Moher et al., 2015). Dozens of journals have endorsed these guidelines, including the Nature publishing group (which publishes *Nature* and *Nat. Neurosci.*), Cell Press (which publishes *Neuron*), *Science*, and *J. Neurosci.* and *eNeuro*. Interestingly, *J. Neurosci.* showed substantial decreases in sex omission even before the convening of the workshop that resulted in the NIH Principles and Guidelines in Reporting Preclinical Research (Fig. 5*A*). This may reflect internal editorial policy, enforcement, and methods presentation. That *J. Neurosci.* has one of the lowest rates of sex omission during the assessed time period, even compared with other journals that successfully decreased sex omission, suggests that the mechanisms by which editorial policies are enforced by an individual journal plays an important role. Studies of the effectiveness of ARRIVE and other guidelines seem to support this speculation (Smidt et al., 2006; Moher et al., 2010; Turner et al., 2012; Baker et al., 2014; Sekula et al., 2017). Thus, the effectiveness of different enforcement techniques across journals should be directly assessed by future studies, especially comparing journals that mandate the inclusion of sex in both the title and methods of manuscripts (Blaustein, 2012); journals that include animal sex is reported on author, reviewer, or editor checklists (Han et al., 2017); journals with statements in the author guidelines; and journals with no relevant policies at all. A significant challenge of understanding the etiology of differences between journals is the temporal lag between the implementation of journal policies and its effects on individual research articles. Given the lengthy time required for manuscript preparation, peer review, and manuscript revision, it may take months or perhaps years for manifestation of changes at the level of editorial or granting agency policy to be reflected in individual research articles. Nevertheless, future studies should continue to monitor sex omission, sex bias, and potentially other critical experimental details across years, especially since the analysis presented here ends in 2014. This would allow for the evaluation of relevant scientific journal policies and help remove the potential barriers to scientific reproducibility generated by erratic reporting of animal sex. This will be particularly important given the emerging recognition that sex can play a significant and complex role in influencing specific neural substrates.

## Synthesis

Reviewing Editor: Rae Silver, Columbia University Decisions are customarily a result of the Reviewing Editor and the peer reviewers coming together and discussing their recommendations until a consensus is reached. When revisions are invited, a fact-based synthesis statement explaining their decision and outlining what is needed to prepare a revision will be listed below. The following reviewer(s) agreed to reveal their identity: Sabra Klein, Jill Becker

This is a timely article regarding the reporting of biological sex in neuroscience research between 2010 and 2014 in six of the premier journals in which neuroscience research is published. The authors find that reporting of sex of rodents used in research is increasing, even though there remains a sex bias to use males more than females. Analysis and reporting of comparison of sex differences is increasing although there are considerable differences across journals and many of the articles reviewed still did not consider sex as a biological variable.

The comments have been categorized as “major” and “minor”. The authors are asked to address each of these or to explain why they have not been considered in the manuscript.

Major:

1. There was no mention of the journal policies associated with sex reporting or possible changes in journal policies during the period from 2011-2014. It is very important to include information on whether any of the journals analyzed had policies regarding sex reporting from 2011-2014, and how that changed the results. This may include, but should not be limited to, mention of sex as a variable in the instructions to authors, inclusion of sex as a variable in an author's checklist, or consideration of sex as a variable in statistical analyses. Without this information, it is difficult to interpret the observed differences across the six journals used in the analysis, as the etiology of differences between journals may reflect differences in sex reporting policies by journals.

2. Consider the definition of sex as a biological variable (SABV). The NIH guidelines require that data are reported for both males and females, not that it is necessary to compare males and females. The authors used the definition of comparison of males and females, whether or not the data or analyses are shown. A subtle, but important, difference if the data reviewed here are to be used to assess the effect of the new SABV guidelines. Can the authors report the data in both ways - i.e. the number of articles in which the data are reported separately for both males and females (even when combined for analyses) and those in which males and females are compared.

3. In the Abstract, Significance statement, and Introduction, it appears that the authors overstate the male bias in biomedical literature. For example, Beery and Zucker 2011 and Klein et al. 2015, indicate that biases are present across a large range of biomedical disciplines, but male-biases are limited to distinct fields, including neuroscience. Thus it is an overstatement to say that all biomedical research suffers from male biases. As in previous analyses, it appears that neuroscience, not all biomedical research, is particularly bad about using predominantly male rodents in preclinical research. (Other fields, including immunology, are particularly bad about using almost exclusively females in preclinical animal studies.) These nuances are important and should either be captured, or gross generalizations should be omitted.

4. The rationale for exclusion of species other than rats and mice in this review is not provided. According to the data presented in figure 1, non-rat and mouse studies account for 50% of all neuroscience animal studies. Given that neuroscience is rich with comparative animal models, why were such a large proportion of studies omitted? Given that no compelling rationale, then data from other species should be included in the analyses---this could become especially valuable if the trends are different from those reported for rats and mice.

Minor:

1. The data presented in Figure 4 are not well graphed. Given that the point is to provide a visual that shows differences across journals in sex reporting, it may be of greater value to show pie charts or single proportion bars for each journal, with the proportions for each category represented. Pie charts (one for each year and each journal) may be an interesting way to show proportional changes over time.

2. The word ‘data’ is plural: e.g. ‘these data’, not ‘this data’.

3. The analysis by impact factor by year is not particularly helpful. There are too few data points (6) each year to provide statistical power. Combining the analyses across years might be more informative - but Fig 5 could also be omitted.

## Author Response

Author point-by-point reply

We appreciate the constructive and helpful comments from the reviewers. After careful consideration of the reviews, we have heavily revised the manuscript. Changes in the manuscript text are indicated with yellow highlighting. Below we provide a point-by-point description of how we addressed each comment. Thank you for considering this resubmitted manuscript for publication in eNeuro. We appreciate the positive comments regarding our manuscript.

The explanations below are numbered to directly correspond to the reviewers' comments.

Major Comments

1. We agree that this is an important component for the Discussion, and as requested we now include this in the manuscript (Page 11, lines 30-31; Page 12-13, all lines). While our study was not designed to elucidate the etiology of differences in sex omission and bias between journals, we hope that this new Discussion section is useful for both interpreting the novel results of the current study, and constructively informing the work of future studies.

2. We agree with the reviewers that this is an important point of discussion, and have now both clarified the methods section (Page 5, lines 7-10) and addressed this point in the Discussion (Page 10, lines 1-27). The dataset does not allow for the distinction between articles in which data are reported separately for both males and females and not statistically compared, versus articles in which male and female datasets were combined and not statistically compared. Anecdotally there were very few articles in which data were reported disaggregated by sex but did not perform some sort of comparison.

3. We agree with the Reviewers' comments and have revised the manuscript to exclude inaccurate gross generalizations (Abstract, lines 2, 14-16; Significance Statement, line 18 and 20; Page 3, lines 4-8, removal of sentence that overgeneralized, and adjusted references; Page 9, lines 4, 12-13).

4. As requested we now provide the rationale for exclusion of species other than rats and mice (Page 4, lines 29-30; Page 10, lines 28-31; Page 11, lines 1-29).

Minor Comments

1. To address this comment we have made two changes to the manuscript. First, as requested we now show pie charts for each journal in 2010 and 2014 (new Figure 4). We agree with the reviewers that this helps increase clarity. We found that showing pie charts for all years was problematic due to the number of necessary pie charts (30!). Thus, for our second change we revised the original figure to increase the size of individual data points, overall figure size, and adjust legend and font sizes. We hope that this two pronged approach successfully addresses the reviewers' concerns by both illustrating the most critical point, i.e., that there are differences in reporting sex across journals (new Figure 4, pie charts), and conveying the wealth of temporal information generated by this study (new Figure 5, revised dot and line graphs).

2. Typo now corrected throughout the manuscript. Thanks much for catching this embarrassing typo.

3. As requested we have removed this analysis from the manuscript.
